# The Efficacy of Pralidoxime in the Treatment of Organophosphate Poisoning in Humans: A Systematic Review and Meta-analysis of Randomized Trials

**DOI:** 10.7759/cureus.7174

**Published:** 2020-03-04

**Authors:** Himal Kharel, Nishan B Pokhrel, Rakesh Ghimire, Zeni Kharel

**Affiliations:** 1 Clinical Pharmacology, Tribhuvan University Institute of Medicine, Kathmandu, NPL; 2 Internal Medicine, Tribhuvan University Institute of Medicine, Kathmandu, NPL; 3 Internal Medicine, Rochester General Hospital, Rochester, USA

**Keywords:** organophosphates, poisoning, pralidoxime, oximes, insecticides

## Abstract

Introduction

The benefits of atropine in the treatment of acute organophosphate (OP) poisoning has been well established, while that of oximes is still uncertain. Pralidoxime is the most often used oxime worldwide. In vitro experiments have consistently shown that oximes are effective reactivators of human acetylcholinesterase enzyme, inhibited by OP compounds. However, the clinical benefit of pralidoxime is still unclear. A recent meta-analysis has found that pralidoxime provides no significant improvement in outcome and rather may cause harm while increasing the economic burden in low-income communities where its use is the most prevalent.

Objectives

This study aimed to provide an updated evaluation of the efficacy of pralidoxime in addition to atropine alone in the treatment of patients with acute OP poisoning in terms of mortality, need for ventilator support, and the incidence of intermediate syndrome. The intermediate syndrome is a clinical syndrome that occurs 24 to 96 hours after the ingestion of an OP compound and is characterized by prominent weakness of neck flexors, muscles of respiration, and proximal limb muscles.

Materials and methods

We searched MEDLINE, EMBASE, CENTRAL, and ClinicalTrials.gov databases until January 2019 for randomized controlled trials (RCTs) in the English language that evaluated the use of pralidoxime in individuals of any age, gender or nationality presenting with an alleged history of OP intake. The primary outcome was mortality. Secondary outcomes were the need for ventilator support and the incidence of intermediate syndrome. The risk of bias in included studies was assessed using the tool recommended by the Cochrane Handbook of Systematic Review of Interventions. Treatment/control differences in these outcomes across included studies were combined using risk ratios (RR).

Results

Six randomized controlled trials (n = 646) fulfilled the inclusion criteria, including one further trial missed from the most recent systematic review. The risk of bias varied across studies, with Eddleston 2009 being of the lowest risk and Cherian 2005 being of high risk. The risk of mortality (RR = 1.53, 95% confidence interval (CI) 0.97 to 2.41, P = 0.07) and the need for ventilator support (RR = 1.29, 95% CI 0.97 to 1.71, P = 0.08) were not significantly different between the pralidoxime and the control group. There was a significant increase in the incidence of intermediate syndrome in the pralidoxime group (RR = 1.63; 95% CI 1.01 to 2.62, P = 0.04).

Conclusions

Based on our meta-analysis of the available RCTs, pralidoxime was not shown to be beneficial in patients with acute OP poisoning. Our findings are consistent with the other literature.

## Introduction

Organophosphates (OPs) are commonly used for pest control in agriculture. The commonly used compounds in developing countries like India and Sri Lanka include methyl parathion, malathion, fenthion, chlorpyrifos, quinalphos, and diazinon [1,2]. The number of intoxications with OP compounds is estimated to be three million per year, and the number of deaths approximately 100,000 per year [3,4]. This predominantly affects low-income countries [5]. OP compounds cause inhibition of acetylcholinesterase (AChE) enzyme through its covalent modification leading to excessive cholinergic stimulation on the nicotinic and muscarinic receptors. Overstimulation of muscarinic receptors results in seizures, excessive secretion, increased bowel and bladder activity with nausea, vomiting, diarrhea, abdominal cramps, incontinence of feces and urine and seizures. Overstimulation of nicotinic receptors results in hypertension, tachycardia, muscle cramps, fasciculations, weakness, and paralysis. Death usually occurs due to respiratory failure. Intermediate syndrome is a distinct clinical entity. It usually occurs 24 to 96 hours after the ingestion of an OP compound after an initial cholinergic crisis, but before the expected onset of delayed polyneuropathy [6]. This syndrome is characterized by prominent weakness of neck flexors, muscles of respiration and proximal limb muscles. Though the exact pathogenesis of intermediate syndrome is unclear, the proposed mechanisms include persistent inhibition of AChE leading to functional paralysis of neuromuscular transmission, muscle necrosis, and oxidative free radical damage to the receptors [7,8].

Pralidoxime (2-PAM) is the most often used oxime worldwide. It has four salts: chloride (2-PAM Cl), methiodide, methyl sulfate, and mesylate (P2S). It is commonly used with atropine but it has to be administered before the aging of the AChE enzyme [9]. Adverse effects of 2-PAM include dizziness, drowsiness, blurred vision, occasional diplopia, impaired accommodation, nausea, headache, tachycardia, hyperventilation, hypertension and muscular weakness due to transient neuromuscular blockade [10].

In vitro experiments have consistently shown that oximes are effective reactivators of OP-inhibited human AChE enzyme [11]. Oximes work by reactivating acetylcholinesterase that has been bound to the OP molecule. Oximes can be highly effective in restoring skeletal muscle strength and improving diaphragmatic weakness where atropine has virtually no effect [3].

The role of pralidoxime in the management of OP poisoning is controversial. Systematic reviews and meta-analyses have consistently found that pralidoxime provides no significant improvement in the outcome and rather may cause harm while increasing the economic burden in low-income communities where it is the most prevalent [5]. A recent meta-analysis by Blumenberg et al. [12] also concluded that pralidoxime does not prevent death or the need for ventilator support. Intending to update previous systematic reviews with new studies, we conducted a meta-analysis of data published through January 2019.

We aimed to clarify the evidence regarding the efficacy of pralidoxime compared with atropine alone in the treatment of patients with organophosphorus poisoning in terms of mortality, the need for ventilator support, and the incidence of intermediate syndrome.

## Materials and methods

Protocol and registration

We have registered our protocol in PROSPERO International Prospective Register of Systematic Reviews where we have pre-specified the objectives and methods of the analysis. The registration number is CRD42019123058.

Eligibility criteria

1. Eligible study types: Prospective randomized controlled trials (RCTs) in the English language were considered eligible to be included in this review. Non-English language and animal studies were excluded.

2. Eligible study participants: Individuals of any age, gender or nationality, with a history of an alleged OP exposure (any form of OP compound).

3. Eligible study interventions: Pralidoxime (other oximes were not included), atropine and supportive care. The dose of pralidoxime and atropine and the quality of the supportive care varied widely across studies.

4. Eligible study comparison: Use of placebo, atropine and supportive care, without the addition of pralidoxime.

5. Types of outcome measures: We predefined mortality as the primary outcome measure and, the need for ventilator support and the incidence of intermediate syndrome as secondary outcome measures.

Information sources

We conducted our search from September 11, 2009 to January 25, 2019. Studies until September 11, 2009 were included in the final Cochrane search [13]. Two reviewers (NBP and ZK) independently searched the MEDLINE, EMBASE, CENTRAL and ClinicalTrials.gov databases using the search strategy to identify RCTs that meet the eligibility criteria. The terms organophosphate poisoning, insecticides, poisoning, and oximes were searched under the medical subject headings (MeSH) terms. The term pralidoxime was searched under the Supplementary Concept. We limited study types to RCTs in the English language and the study subjects to humans. In addition to these, the references of previously published reviews were checked to identify studies that were not found in the previous databases. Further, the reference lists of trials qualifying in this study were checked to identify further studies not found in the previous database searches. Experts in this field were also contacted for any information regarding additional or ongoing studies they might have. Lastly, unpublished studies were searched using OpenGrey, GreyLit and the Grey Matters databases.

Study selection

Studies were selected using the inclusion criteria. The titles and abstracts of the references retrieved during the searches were screened in duplicate by two reviewers (NBP and ZK), and potentially relevant full-texts were then screened in pairs by authors.

Data extraction

Data were abstracted independently by two reviewers (NBP and HK) from the studies selected for inclusion onto a standardized Excel form which was pilot tested and refined. Discrepancies were resolved through discussion with other team members. The extracted data included: year, country, study design, total number of participants randomized, setting, diagnostic criteria, inclusion and exclusion criteria, the severity of poisoning, the time of initiation of pralidoxime, mean age, sex, outcomes, dose and disposition of patients.

Risk of bias in individual studies

Three reviewers (NBP, HK, and RG) independently assessed the risk of bias for each included study using a specific tool recommended by the Cochrane Handbook for Systematic Reviews of Interventions. This tool addresses a specific feature of the study as an entry in a "Risk of bias" table. Each of the following entries seeks the presence of the respective bias in the included study: random sequence generation (selection bias), allocation concealment (selection bias), blinding of participants and personnel (performance bias), blinding of outcome assessment (detection bias), incomplete outcome data (attrition bias), selection reporting (reporting bias) and other biases.

Each entry in this table had its description and judgment. The judgment for entry involved answering a question, with answers ‘Yes’ indicating a low risk of bias, ‘No’ indicating a high risk of bias, and ‘Unclear’ indicating either lack of information or uncertainty over the potential for bias. After doing this, a plot of ‘risk of bias’ assessments was created in the Review Manager software (version 5.3). Then the reviewers rated each study by counting the total number of ‘low risk’ in the ‘authors’ judgment’ column of the ‘risk of bias’ table. As there were seven entries in total, the total number of ‘low risk’ a study can get was seven. So, each study received a rating out of seven. Discrepancies were resolved through consensus. None of the reviewers was blinded to the identity of study authors or study results.

Summary measures

For all outcomes, we extracted the total number of patients in each group and the number of patients experiencing an event. Risk ratios (RRs) with 95% confidence intervals (CIs) were calculated as the primary outcome measures. All included studies carried out randomization at the participant level. We considered the unit of analysis as each patient recruited to a trial. All the included studies provided data required for the analysis. One study lacked data on age and gender for which we made a failed attempt to contact the author [14].

Synthesis of results

The primary measure of interest was mortality and the secondary measures were the need for ventilator support and the incidence of intermediate syndrome. Treatment/control differences in these outcomes across included studies were combined using risk ratios (RR). Data analysis was done using the Review Manager software (version 5.3). A random-effects model was used because there was variance in the study setting, the type of OP compound exposed and the dosing.

We have assessed between-study heterogeneity using Cochrane’s Q test and by estimation of the percentage heterogeneity between studies that cannot be ascribed to sampling variation with the I^2^ statistic along its 95% CI using restricted maximum likelihood estimator. We considered heterogeneity to be substantial if the I^2^ statistic exceeded 50%. Forest plots were also visually inspected.

Risk of bias across studies

Though we intended to assess publication bias, it was not looked for as there was insufficient power to properly assess a funnel plot or more advanced regression-based assessments.

Additional analyses

Irrespective of heterogeneity, subgroup effects can be present [15]. Subgroup analyses were done based on the dose of pralidoxime administered, disposition of patients and the severity of poisoning. We performed sensitivity analyses by removing studies that we considered to be at a high or unclear risk of bias and examined any change in the summary statistic.

## Results

Study selection

**Figure 1:**
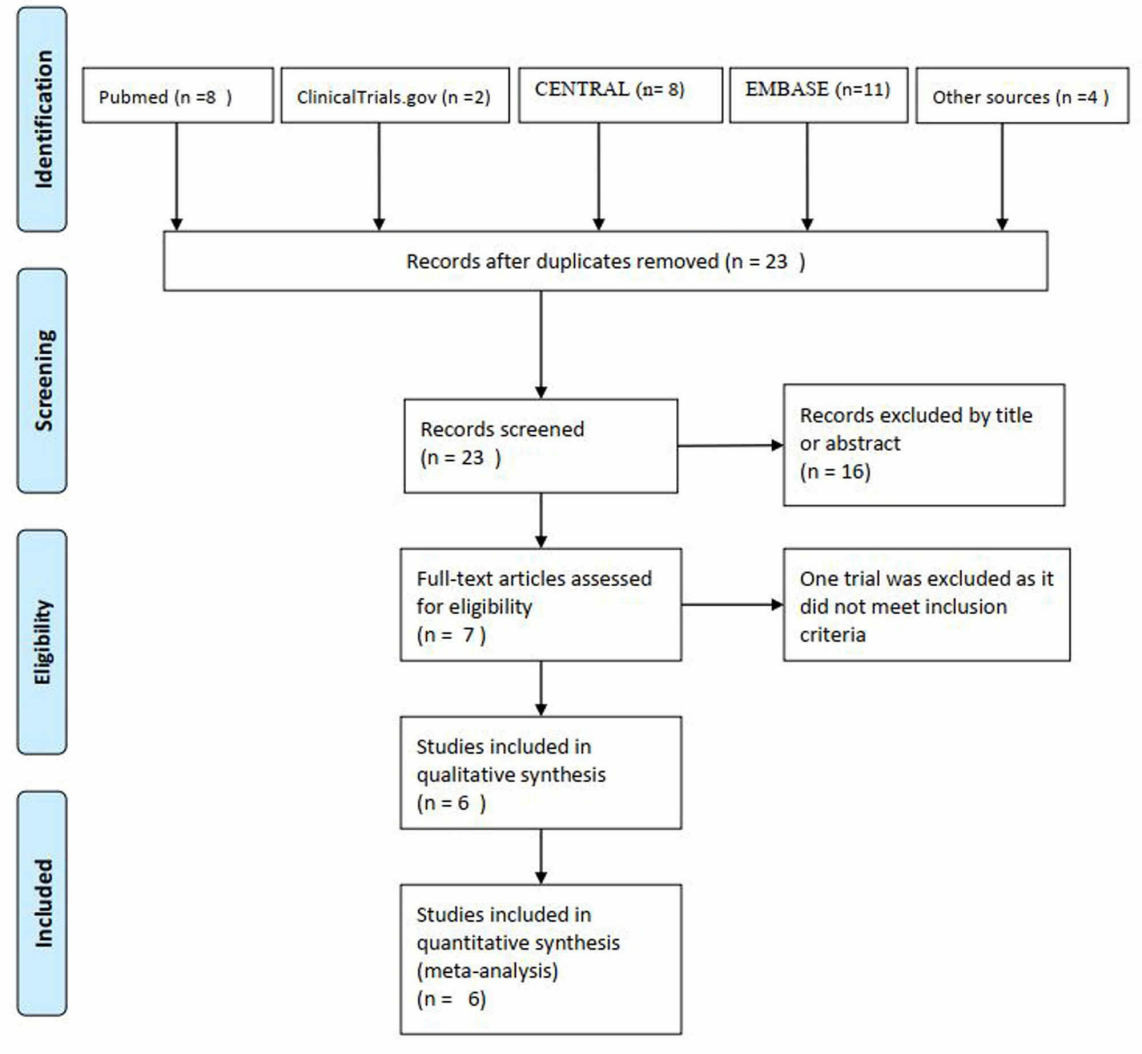
Flow diagram of the study selection process

We identified 23 articles from our search of which three articles were added from Cochrane Review by Buckley et al. and another article from the reference list of an RCT (Figure 1) [13,14,16-19]. Seven full-text articles were retrieved for detailed evaluation. In total, six RCTs enrolling 646 participants and satisfying the eligibility criteria were finally analyzed [14,16-20].

Study characteristics

The included studies varied in their individual inclusion and exclusion criteria, and the dose of pralidoxime administered. The duration of poisoning before arrival to the hospital also varied across studies. While some studies were not so rigid in the time of presentation, others included only those cases, if they presented before 12 hours, before 24 hours, and before 48 hours [14,16-20]. Some studies did not define the age limit [14,16]. While others strictly included those more than 12 years of age or more than 14 years [17-20]. Only the cases of self-poisoning were included by Eddleston et al., whereas, all other studies included intentional and unintentional poisoning [14,16-20]. All the studies except Cherian et al. and Eddleston et al. excluded the cases with carbamate poisoning (while noting that it was impossible to exclude carbamate poisoning based on clinical presentation and history alone, as performed in those studies) [14,16-20]. Half of the included studies had specified the type of OP compound [17,19,20]. Similarly, only three studies excluded pregnant patients [17-19]. Some studies also defined the severity of poisoning while enrolling in the subjects. Cherian et al. and Syed et al. included only the cases with moderate to severe poisoning [14,16,20]. Eddleston et al. excluded patients who did not require atropine thereby excluding mild/asymptomatic poisoning [17]. No such specificity was mentioned in other studies [18,19]. The administered dose and frequency of pralidoxime varied across studies. Dosing of atropine also varied across studies but all of them aimed to achieve and maintain the signs of atropinization (clear chest on auscultation with no wheeze, heart rate >80 beats/min, pupils no longer pinpoint, dry axillae and systolic blood pressure >80 mmHg). Cherian et al. titrated the dose of atropine to maintain atropinization [14,16]. However, no specific dose was mentioned. Eddleston et al. and Syed et al. used 1.8-3 mg intravenous bolus initially, doubling every five minutes until atropinization was reached following standard guidelines [17,20-22]. It was then infused to maintain the blood atropine concentration in the therapeutic range. Banerjee et al. gave 2 mg intravenous bolus initially and then 2 mg intravenous every 5-10 minutes until the atropinization was achieved (Table 1) [18,19].

**Table 1:** Characteristics of RCTs included in this meta-analysis C: Control group, I: Intervention group, ICU: Intensive Care Unit, IMS: Incidence of an intermediate syndrome, IQR: Inter-quartile range, M: Mortality, n: Sample size, PAM: Pralidoxime SD: Standard deviation, V: Requirement of ventilator support. RCTs: Randomized controlled trials.

Study	Country	Patients (n)	Males (%)	Age (mean ± SD)	Intervention (I) and comparison (C)	Outcomes (I/C)	Disposition
Cherian et al. [16]	India	110 I: 55, C: 55	I: 41 (75), C: 34 (62)	I: 28 ±10.1, C: 26.5 ± 10.3	I: PAM infusion of 12 grams over 3 days C: Normal saline infusion for 3 days	M: 16/3, V: 37/22, IMS: 36/19	ICU only
Cherian et al. [14]	India	21, I:10, C: 11	NA	NA	I: PAM infusion of 12 g/day for 3 days in severe cases and 4 g/day for 3 days in moderate cases C: Normal saline infusion	M: 1/1, V: 7/4,	ICU only
Eddleston et al. [17]	Sri Lanka	235, I: 121, C: 114	I: 96 (79.3), C: 92 (80.7)	I: 31 ± 4.3, C: 29.5 ± 3.2	I: 2g loading dose over 20 min, then a constant infusion of 0.5g/h until 7 days, atropine had not been required for 12-24 h or death C: Normal saline infusion	M: 30/18, V: 26/24,	ICU and general wards
Banerjee et al. [18]	India	60, I: 30, C: 30	I: 14 (47), C: 11 (37)	I: 34.6 ± 9.8, C: 34.3 ± 8.8	I: PAM in a dose of 0.5-1 g 6 hourly, C: Atropine only	M: 2/1, V: 6/2	ICU and general wards
Banerjee et al. [19]	India	120, I: 60, C: 60	I: 23 (38), C: 26 (43)	I: 34.6 ± 9.8, C: 34.3 ± 8.8	I: PAM in a dose of 1 g every 6 hours for a period of 5 days, C: Atropine alone	M: 11/8, V: 3/5	ICU and general wards
Syed et al. [20]	India	100, I: 50, C: 50	I: 19 (38), C: 20 (40)	I: 29.1 ± 10.9, C: 28.2 ± 9.9	I: PAM at 30 mg/kg loading dose over 30 min followed by 8 mg/kg/h continuous infusion for a maximum of 7 days, C: Normal saline	I: 13/14, V: 31/29, IMS: 10/9	ICU only

Risk of bias within studies

**Figure 2:**
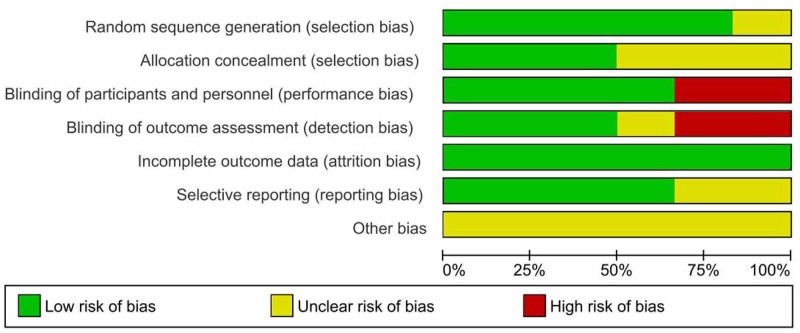
Risk of bias graph: review authors' judgment about each methodological quality item presented as percentages across all included studies

**Figure 3:**
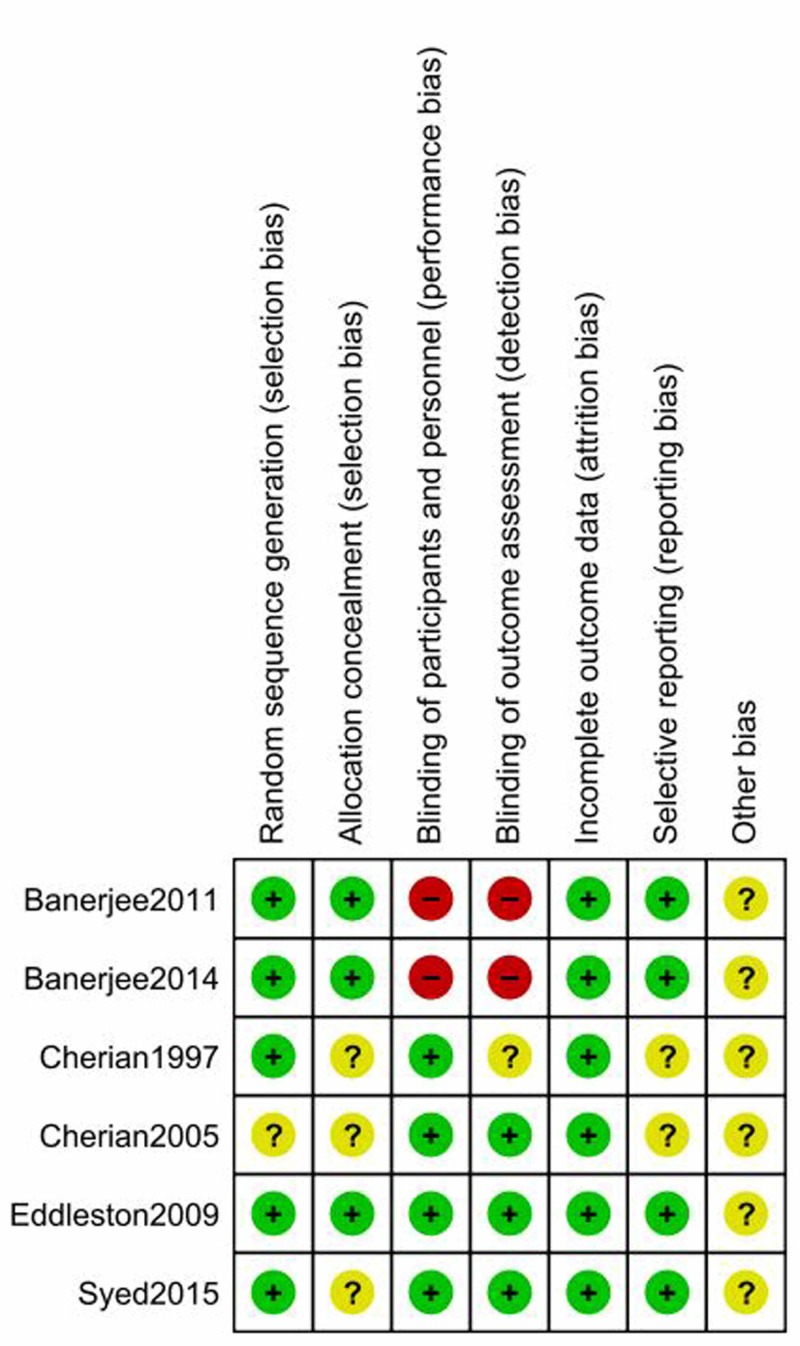
Risk of bias summary: review authors' judgment about each methodological quality item for each included study

The graphical summaries of the 'Risk of bias' assessment for the six included studies are provided. (Figures 2-3). The intra-class correlation coefficient of 0.97 (95% CI 0.889-0.996) reflects the considerable agreement among the reviewers.

1. Allocation: Some studies did not mention the information regarding random sequence generation or allocation concealment [14,16,20]. Two studies used a block randomization schedule to randomize the patients [16,17]. Others used the computer-generated random allocation sequence for the recruitment of participants [18-20].

2. Blinding: Blinding was not done in two studies, while it was still unclear in another study [16,18,19].

3. Incomplete outcome data: All studies reported outcomes on all randomized patients. All studies had a low risk of attrition bias or reporting biases.

4. Selective reporting: Three studies were pre-registered in trial registries and reported all pre-specified outcomes and adverse outcomes [17,20]. Among those registered, Banerjee 2014 was registered after the trial completion [19]. Others were not registered [14,16,18]. Banerjee 2011 did not mention outcome variables [18].

5. Other potential sources of bias: Most of the studies (except Eddleston 2009) did not calculate the sample size and power, leading to the high risk of other biases [17]. These studies did not mention the plan for interim analysis or stopping rules. Unfortunately, Eddleston 2009 was prematurely stopped due to publication of a trial of high vs low dose pralidoxime with an unexpectedly large effect of high dose pralidoxime on all outcomes which resulted in a loss of clinician equipoise [17,23].

Results of individual studies

1. Primary Outcome: Mortality

**Figure 4:**
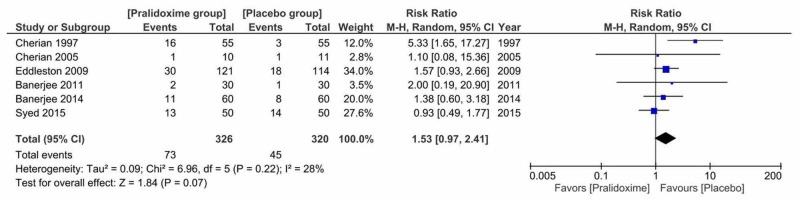
Pralidoxime vs placebo, primary outcome: mortality

All of the six included studies reported mortality among 646 patients. The mortality rate was 22.4% (73 out of 326) in the pralidoxime group and 14.1% (45 out of 320) in the control group. No effect of oxime therapy was observed on overall mortality (RR = 1.53, 95% CI 0.97 to 2.41, P = 0.07). Cochran's Q test suggested that the studies were not significantly heterogeneous (I^2^ = 28% and P = 0.22) (Figure 4).

We also studied the effect of pralidoxime versus placebo on mortality by performing a sensitivity analysis to investigate the effect of removing studies which had high risk of selection bias [14,16,20]. We found that this did not significantly alter the findings (RR 1.53, 95% CI 0.99 to 2.36; $\chi ^{2}$ = 0.12, df = 2(P = 0.94); I^2^ = 0%; 3 trials, N = 415; random-effects).

Subgroup analyses were performed based on the dose of pralidoxime, disposition of patients and the severity of poisoning. The dose of pralidoxime was separated into two groups: the group receiving WHO dose or WHO equivalent dose (30 mg/kg then 8 mg/kg/hr infusion or 2 g loading dose followed by 0.5 g/hr infusion) and the group receiving sub-WHO doses (less than 12 g/day). There were no statistically significant differences between subgroups for a dose of pralidoxime, disposition of patients and the severity of poisoning.

2. Secondary Outcome: The Need for Ventilator Support

**Figure 5:**
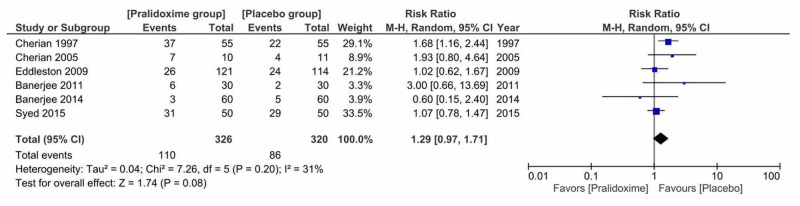
Pralidoxime vs placebo, secondary outcome: the need for ventilator support

All of the six included studies reported the need for ventilator support among 646 patients. Ventilator support was required in 33.7% (110 out of 326) of patients treated with pralidoxime and 26.9% (86 out of 320) in the control group. There was no effect of pralidoxime therapy on the need for ventilator support (RR = 1.29, 95% CI 0.97 to 1.71, P = 0.08). Cochran's Q test suggested that the studies were not significantly heterogeneous (I^2^ = 31% and P = 0.20) (Figure 5).

We also studied the effect of pralidoxime versus placebo on the need for ventilator support by performing a sensitivity analysis to investigate the effect of removing studies that had a high risk of selection bias [14,16,20]. We found that this did not significantly alter the findings (RR = 1.09, 95% CI 0.59 to 2; $\chi ^{2}$ = 2.48, df = 2(P = 0.29); I^2^ = 19%; 3 trials, N = 415; random-effects). There were no statistically significant differences between subgroups for a dose of pralidoxime, disposition of patients and the severity of poisoning.

3. Secondary Outcome: The Incidence of Intermediate Syndrome

**Figure 6:**
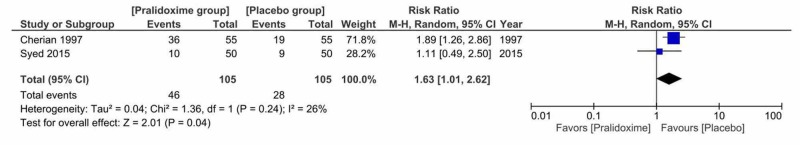
Pralidoxime vs placebo, secondary outcome: the incidence of intermediate syndrome

Only two studies reported the incidence of intermediate syndrome among 210 patients. Intermediate syndrome appeared in 43.8% (46 out of 105) of patients in the pralidoxime group and 26.7% (28 out of 105) in the control group. Pralidoxime therapy was associated with a significant increase in the incidence of intermediate syndrome (RR = 1.63; 95% CI 1.01 to 2.62, P = 0.04). This indicated slightly increased risk of intermediate-risk in the pralidoxime group. Cochran's Q test suggested that the studies were not significantly heterogeneous (I^2^ = 26%, P = 0.24) (Figure 6).

## Discussion

Pooled trials suggested that pralidoxime was not beneficial to patients with OP poisoning in terms of mortality, the need for ventilator support and the incidence of intermediate syndrome. Among these three outcomes, a significant result was obtained only for the incidence of intermediate syndrome, with a higher risk of intermediate syndrome in the pralidoxime group. Though it was not statistically significant, we noticed a trend to an increase in mortality (P = 0.07) and the need for ventilator support (P = 0.08) in the pralidoxime group. The test of interaction between the two subgroups based on the dose of pralidoxime administered, the disposition of patients and the severity of poisoning showed no significant differences in mortality or the need for ventilator support. No significant alteration in findings was observed when we included only those studies with a low risk of selection bias.

We extensively searched all relevant studies to this review and found six studies randomizing 646 participants to either of pralidoxime or placebo groups. Among them, only one was at a low-risk summary of bias [17]. Trials were published between 1997 and 2015 and took place in two South Asian developing countries (India and Sri Lanka). The duration of poisoning before the presentation varied across studies. Some of them also included cases with carbamate poisoning [14,17]. Most of them didn’t consider the type of OP compound or the time of administration of oximes. The oximes were also administered in a wide range of doses and durations. Dosing of atropine also varied across studies but all of them aimed to achieve and maintain the signs of atropinization. All the studies examined the primary outcome ‘mortality’ and a secondary outcome 'need for ventilator support' and two included studies examined the ‘incidence of intermediate syndrome’.

Various in vitro experiments, ex vivo observational studies and an RCT demonstrated that oximes reactivate the AChE enzyme which has been inhibited by OP compound [17,24,25]. The beneficial effect of oximes had been proven in animals and the optimum dose been identified, which was later extrapolated in humans [26]. However, similar efficacy has not been demonstrated clinically. Thiermann et al. noticed a long time for the reactivation of the enzyme in vivo than expected from in vitro experiments [25]. Interestingly, people were found to have survived despite high degrees of AChE inhibition [17]. In their study, Worek et al. found marked differences in the reactivating potency of oximes in different animal species [11]. So, the available animal data about oximes may not be adequate to fully explain their effects in humans.

Most of the cases studied in the trials were of intentional poisoning for self-harm for which a person ingested a large amount of poison. Ingestion of such a large poison load results in an overwhelming concentration of OP within the patient, which persists in the body for a longer period and which rapidly inactivates the newly reactivated or formed enzyme [24,25]. To counter this high poison load, a high concentration of pralidoxime is required which failed to show any benefits [17].

A small retrospective Sri Lankan study found no significant difference in the outcomes in acutely OP poisoned patients treated only with atropine when pralidoxime was temporarily out of supply [1]. Many researchers believed that the dose of pralidoxime was too low to be effective [24,27]. Worek et al. asserted that the usual recommended dose of 4 micro g/mL was not enough to reactivate all the inhibited AChE [24]. So, a higher dose was recommended by WHO to maintain the adequate plasma concentration of pralidoxime [27].

Even that high dose regimen was not found to be effective so far. Only an Indian study reported in favor of this high dose regimen in which a reduction in mortality was observed with high dose pralidoxime (1 g/h) compared to a low dose regimen (1 g every four hours) administered for 48 hours [23]. Contrary to this, the other three studies in which high dose pralidoxime infusions were administered for a maximum of seven days demonstrated no beneficial effect in pralidoxime groups [14,17,20]. Eddleston et al. rather reported a 69% increase in mortality due to the treatment with pralidoxime [17]. These facts suggest that insufficient dosing was not responsible for the absence of an effect.

These studies were not free from limitations. Two of them included only intensive care unit (ICU) patients [14,20]. However, contrary to the possibility of recruiting sicker patients to the treatment arm as pointed out by Buckley et al., we found no significant differences in the baseline characteristics of the two groups (Table 2) [13].

**Table 2:** Baseline characteristics of the two treatment groups DM OP: Dimethyl organophosphate poisoning; severe poisoning: severe grade defined by the authors or by the presence of coma. Unpaired t-test used for continuous variables; a: categorical result by Fisher’s exact.

Characteristic	No. of studies	Pralidoxime group	Control group	P- value
No. of patients	6	326	320	
Age, mean (SD)	5	31.5 (3.1)	30.6 (3.6)	0.68
Males/females in included studies	5	193/123	183/126	0.91/ 0.75^a^
No (%) of patients with DM OP poisoning	3	98 (43.8)	103 (48.5)	0.68^a^
No (%) of patients with DE OP poisoning	3	95 (38.7)	87 (36.6)	0.93^a^
No (%) of patients with severe poisoning	6	162 (49.7)	155 (48.4)	0.66^a^
Average pseudocholinesterase levels, mean (SD) IU/L	2	1400.1 (589.5)	1940.6 (320.5)	0.64
Average butyrylcholinesterase levels, mean (SD) IU/L	2	972.3 (565.9)	823.6 (426.8)	0.90

Knowing about the class of an OP compound is imperative because dimethyl and diethyl group of compounds differ completely in their ageing and reactivation kinetics. Dimethyl inhibited AChE enzyme reactivates and ages quickly, while these processes are slow for diethyl compounds [24]. In patients presenting more than 12 hours of exposure to a poison, they would be benefitted only if the ingested compound was diethyl OP [28]. Dimethyl OP would already have aged and it would be impossible for pralidoxime to reactivate it.

Regional factors in India and Sri Lanka need to be considered as all of the included studies were conducted in the same region. The common OP compounds in India and Sri Lanka are dimethyl compounds like methyl parathion, malathion, fenthion, etc., and diethyl compounds like chlorpyrifos, quinalphos, and diazinon [1,2]. These poisons are poorly regulated and are easily available. Similarly, some of these compounds being highly lipophilic cause the re-inhibition of AChE for many days as they are being mobilized from fat stores, thus increasing the severity [27]. The poison load is high in suicidal self-poisoning. Most present to the hospital after a substantial amount of time has passed due to the poor availability of transport facilities. By this time, a substantial proportion of the enzyme would already have aged due to which pralidoxime would be unable to reactivate them.

The effectiveness of pralidoxime is also decreased due to the overwhelming concentration of poison. In the setting of the ingestion of a large poison load, the co-formulants (solvents and surfactants) in the bottle might be responsible for substantial non-OP toxicity which is resistant to AChE reactivation [29]. Besides, phosphoryl oximes (POXs) form undoubtedly at physiological enzyme concentrations which have high anticholinesterase activity. This compound is degraded by the POX-hydroxylase enzyme, the activity of which varies among individuals. This variation might have contributed to the varying response to oxime therapy in patients with acute OP poisoning [30].

These results support the conclusions of the previous reviews by Blumenberg et al. with additional data from Banerjee et al. study [12,18]. Pralidoxime may work when administered at the right time and appropriate dose. Probably, the treatment needs to be individualized taking into account the severity of poisoning symptoms, type and amount of OP compound ingested and the time elapsed since the poisoning. Routine high dose pralidoxime regimen (30 mg/kg pralidoxime chloride bolus followed by 8 mg/kg/hr infusion) as recommended by WHO is not supported by studies until now [27].

Some limitations of this study are acknowledged. None of the included studies were free from bias. Among the six included studies, only one study was assessed as low risk of bias for almost all domains (six out of seven). We did not look for publication bias because of the inadequate number of included studies. All the included studies were primarily conducted in developing countries where there were inadequate intensive care facilities. Besides, commonly available OP compounds in those settings are hardly available to the people of the developed world because of tight regulations. Children under 14 years were not included in these trials as well. Hence, the findings of this review may not be generalizable to children and the people of developed countries. As we have only studied the effect of pralidoxime in acute OP poisoning, the conclusion should not be generalized to other oximes.

## Conclusions

The available evidence regarding the mortality and the need for ventilator support is not adequately strong to draw definite conclusions regarding the use of pralidoxime in routine practice. Various factors need to be considered while treating a patient with acute OP poisoning. The treating physician should consider the severity of poisoning symptoms, type and amount of OP compound ingested and the time elapsed since poisoning before treating a patient with acute OP poisoning. Determinations of patient subgroups likely to be benefitted with pralidoxime and the optimal dose are crucial to maximize patient safety and therapeutic efficacy. Future RCTs with robust designs are necessary with stratification of patients according to the severity of presentation, the time elapsed between exposure and treatment, class of OP compound (dimethyl or diethyl), RBC AChE activity, and the potential for ex-vivo reactivation.
